# Design, synthesis, and evaluation of 3,7-substituted coumarin derivatives as multifunctional Alzheimer’s disease agents

**DOI:** 10.1080/14756366.2021.1913137

**Published:** 2021-07-19

**Authors:** Sheunopa C. Mzezewa, Sylvester I. Omoruyi, Luke S. Zondagh, Sarel F. Malan, Okobi E. Ekpo, Jacques Joubert

**Affiliations:** a Department of Pharmaceutical Chemistry, School of Pharmacy, University of the Western Cape, Bellville, South Africa; b Department of Medical Biosciences, University of the Western Cape, Bellville, South Africa

**Keywords:** Alzheimer’s disease, coumarin, cholinesterase, monoamine oxidase, neuroprotection

## Abstract

Multitarget directed ligands (MTDLs) are emerging as promising treatment options for Alzheimer’s disease (AD). Coumarin derivatives serve as a good starting point for designing MTDLs due to their inherent inhibition of monoamine oxidase (MAO) and cholinesterase enzymes, which are complicit in AD’s complex pathophysiology. A preliminary series of 3,7-substituted coumarin derivatives were synthesised and evaluated for enzyme inhibitory activity, cytotoxicity as well as neuroprotective ability. The results indicated that the compounds are weak cholinesterase inhibitors with five compounds demonstrating relatively potent inhibition and selectivity towards MAO-B with IC_50_ values between 0.014 and 0.498 hx00B5;µM. Significant neuroprotective effects towards MPP^+^-compromised SH-SY5Y neuroblastoma cells were also observed, with no inherent cytotoxicity at 10 µM for all compounds. The overall results demonstrated that substitution of the phenylethyloxy moiety at the 7-position imparted superior general activity to the derivatives, with the propargylamine substitution at the 3-position, in particular, displaying the best MAO-B selectivity and neuroprotection.

## 1. Introduction

Neurodegenerative diseases (NDs) are a group of cognitive and movement-related disorders resulting from neuronal loss in the brain. Alzheimer’s disease (AD) is the most prevalent ND with over 30 million diagnosed patients worldwide^1^^,^^2^. Its symptoms are progressive and irreversible, initially characterised by recurring short-term memory loss which progresses to degeneration of higher cognitive functions such as decision making and language. Due to the nature of these symptoms, the disease comes at a great burden economically and socially^3^^,^^4^. There is no singular cause or process that is responsible for this neurodegeneration, rather it is the result of different interlinked mechanisms, underpinned by genetic and environmental factors. This complex pathophysiology has made treatment difficult and only symptomatic relief is provided by the drugs currently available on the market^5–7^.

**Figure 1.**
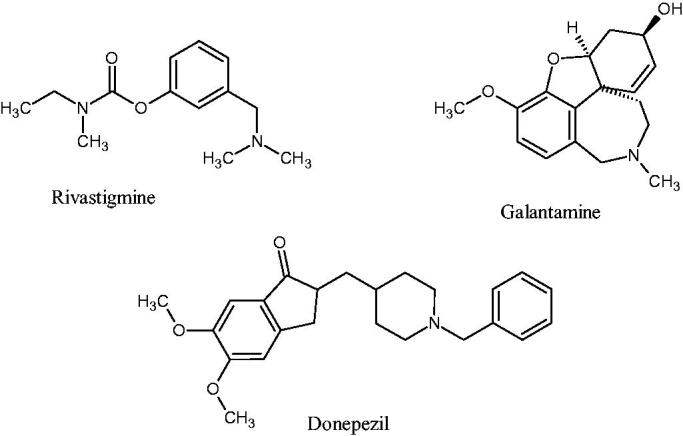
Commercial AChEIs approved for the treatment of AD.

Of the multiple theories underlying AD’s pathophysiology, the roles of oxidative stress, toxic amyloid-β (Aβ) plaques, and the cholinergic hypothesis have gained momentum in explaining the disease’s pathology and in guiding treatment options^8–10^. The cholinergic hypothesis postulates that the symptoms present in AD (particularly cognitive decline and amnesia) can be linked to declining function in the cholinergic neurotransmitter system. The cholinergic system is modulated by the neurotransmitter acetylcholine, metabolised by acetylcholinesterase (AChE) and butyrylcholinesterase (BuChE) in the brain^11^^,^^12^. The primary therapeutic approach has been to counter cholinergic depletion using acetylcholinesterase inhibitors (AChEIs). This has thus far been done by using the second-generation AChEIs such as donepezil, galantamine, and rivastigmine (Figure 1)^5^^,^^13^. The clinical application of these agents has demonstrated that cholinesterase inhibition leads to modest improvement in cognitive functions^14–16^.

Monoamine oxidase (MAO) is a membrane-bound flavoenzyme responsible for metabolising a variety of substrates throughout the body such as serotonin, dopamine, and benzylamine. It exists in two forms, namely MAO-A and MAO-B, with each isoform having differing substrate selectivity and distribution throughout the body. The MAO-B enzyme is the major isoform found in the brain, responsible for 80% of MAO activity in the brain. MAO-B has been implicated in the generation of neurotoxic free radicals and reactive oxygen species which trigger neuroinflammation and the apoptotic cascade in neurons^17^^,^^18^. Studies have found that MAO-B enzyme activity increases with old age and correlates closely with disease progression, making it a target for AD treatment^19–21^.

Dual inhibition towards the causative MAO and cholinesterase enzymes has been demonstrated to not only improve cognitive functions but also to achieve neuroprotection and subsequently halt disease progression^19^^,^^21^. Multitarget directed ligands (MTDLs) provide researchers with a way to use a single drug agent to simultaneously target the mentioned set of enzymes. MTDLs are made by combining pharmacophoric groups with differing pharmacological activities into one molecule to achieve a synergistic therapeutic effect, whilst avoiding the pitfalls of polypharmacy. MTDLs are being explored for use in a variety of complex diseases such as cancer and malaria^7^^,^^22^. Several promising MTDLs have been explored for use in AD including ASS234 and ladostigil which are based on the dual inhibition of MAO and cholinesterases^23^^,^^24^.

**Figure 2.**
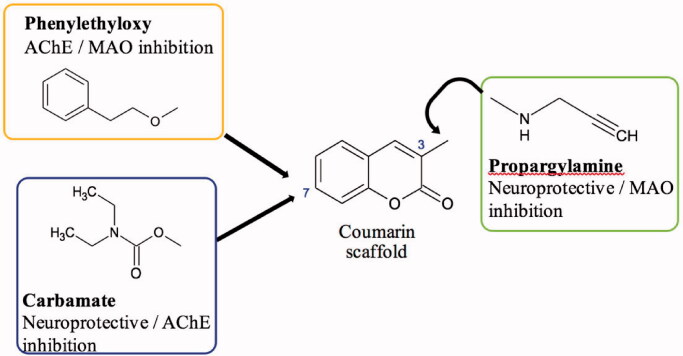
Design strategy of the new coumarin derivatives in this study.

The coumarin scaffold has many intrinsic pharmacological properties and coumarin derivatives have shown promise as a starting point for MTDL design strategies due to its ease of functionalisation and inherent ChE and MAO inhibitory capacity^25–27^. This study, therefore, aimed to further explore the potential of coumarin derivatives as MTDLs by designing and synthesising a small series of compounds that have substitutions of moieties known to enhance inhibition of the MAO and cholinesterase enzymes (Figure 2).

One such moiety is the benzyloxy moiety that has been found to enhance the selectivity and potency of the coumarin scaffold towards MAO-B^28–30^. To optimise the coumarins’ activity towards the target enzymes, a phenylethyl moiety instead of the benzyloxy moiety was attached to the coumarin scaffold in this case. This would ensure that the designed compounds are sufficiently elongated to potentially interact with AChE’s peripheral anionic and catalytic binding sites (also known as dual-site binding), whilst preserving MAO-B binding interactions^31^.

Similarly, the incorporation of the carbamate moiety onto the coumarin scaffold was investigated as well for this study. Numerous studies have found that it confers significant ChE inhibitory effect into various molecules which incorporate it, including the promising MTDL ladostigil^24^^,^^32–34^. These selected moieties were to be incorporated at the 7-position of the scaffold, as substitutions at this position are most beneficial for MAO-B selectivity and inhibition^29^^,^^35^^,^^36^.

Propargylamine, a moiety known for its capacity to confer neuroprotection and increase MAO inhibition was also selected to be substituted at position 3 to enhance the pharmacological profiles of the 7-substituted coumarins^37^^,^^38^. Position 3 of the scaffold has previously been described to be beneficial in achieving dual-site binding in AChE’s active site, thus substitutions at this position would serve an important role in producing dual ChE/MAO inhibitors^38^^,^^39^. These molecules (Figure 2) could have the potential to relieve the symptoms of AD, slow down disease progression and ultimately reduce the burden of the disease.

## 2. Materials and methods

### 2.1. Chemistry

#### 2.1.1. General

All reagents used in the synthesis of the compounds were obtained from Sigma-Aldrich^®^ (Steinheim, Germany) and Industrial Analytical (RSA). All solvents used for reactions and chromatography were purchased from various commercial sources and unless mentioned used without further purification. All reactions were monitored for impurities and completion using thin layer chromatography performed on sheets of 0.20 mm thick aluminium silica gel (TLC Silica gel 60 F245 Merck KGaA) with various mobile phases using volume:volume ratios. Once the plates were developed, the spots were visualised using UV light (254 nm and 366 nm) or iodine vapour. Purification of the specified compounds was performed in glass columns filled with appropriate amounts of silica gel (0.063–0.200 mm/70–230 mesh ASTM, Macherey - Nagel, Duren, Germany) as the stationary phase and with mobile phases indicated for each compound. Nuclear magnetic resonance (NMR) spectroscopy, Fourier Transform Infra-red (FT-IR) spectroscopy, and High-resolution mass spectrometry (HR-MS) techniques were used to elucidate the structures of the compounds. ^1^H and ^13^C NMR spectra were obtained using a Bruker Avance III HD Nanobay 400 MHz spectrometer (Rheinstetten, Germany) equipped with a 5 mm BBO probe at 333 K using standard 1 D NMR pulse sequence. Tetramethylsilane (TMS) was used as internal standard and all chemical shifts are reported in parts per million (ppm) relative to its signal (*δ* = 0) in either deuterated dimethyl sulphoxide (DMSO-d_6_) or deuterated chloroform (CDCl_3_). The following abbreviations are used to describe the multiplicity of signals: s – singlet; d – doublet; dd – doublet of doublets; t – triplet; m – multiplet. The IR data was obtained using a Perkin Elmer Spectrum 400 spectrometer (Waltham, USA) fitted with a diamond attenuated total reflectance (ATR) attachment linked to a computer system. HR-MS data were obtained using a Waters API Q-TOF Ultima spectrometer (Wilmslow, UK) using the electrospray ionisation technique. Melting points were determined by using a Lasec^®^ Melting Point SMP 10 apparatus (Staffordshire, UK) and glass capillary tubes. All experiments involving the use of microwave-assisted methods were performed using a CEM Discover™ closed vessel system (Buckingham, UK). The settings used were custom and specific for each experiment.

#### 2.1.2. Synthesis of 7-hydroxy-3-methyl-2H-1-benzopyran-2-one (1)

A mixture of 2,4-dihydroxybenzaldehyde (7.2 mmol, 1.0 g), sodium propionate (15.6 mmol, 1.5 g), propionic anhydride (19.4 mmol, 2.5 ml), and piperidine (1 mmol, 0.1 ml) was refluxed for 6 h and then poured onto ice. The aqueous mixture was made acidic with 20 ml of a 0.1 N solution HCl and yielded a precipitate that was filtered and treated under stirring with concentrated H_2_SO_4_ (2 ml). The resulting mixture was poured into ice again to afford a red-brown precipitate of the desired product **1**.

Physical Properties: Yield: 60%. mp: 165 °C. ^1^H NMR (400 MHz, DMSO-d_6_): *δ* 7.72 (s, 1H), 7.42–7.40 (d, 1H, *J* = 8.5 Hz), 6.76–6.73 (dd, 1H, *J* = 2.2, 8.4 Hz), 6.69–6.68 (d, 1H, *J* = 2.1 Hz), 2.02 (s, 3H). ^13^C NMR (100 MHz, DMSO-d_6_): *δ* 161.8, 160.2, 154.5, 140.1, 128.7, 120.0, 113.0, 111.8, 101.9, 16.5. HR-ESI [M + H]^+^: calc. 175.0395, exp. 175.0398. FT–IR (ATR): *ν*_max_ (cm^−1^) 3234, 2934, 1739, 1675, 1451.

#### 2.1.3. Synthesis of 3-methyl-7-(2-phenylethoxy)-2H-1-benzopyran-2-one (2)

A microwave-compatible glass-vessel was charged with 200 mg (0.71 mmol) of 7-hydroxy-3-methyl-2H-1-benzopyran-2-one (**1**), 68 mg (2.8 mmol) NaH (80% dispersion in oil), and dissolved in 10 ml of acetonitrile. To this mixture, 570 µl (4.2 mmol) of 2-bromoethylbenzene was added dropwise and subsequently stirred at 80 °C for 5 h under microwave irradiation (maximum power = 150 W). The solvent was removed *in vacuo* and the resulting crude mixture was washed with 30 ml ethyl acetate and 15 ml water. The organic layer was washed with 15 ml of 1 M KOH and then 15 ml of brine, thereafter the solvent was removed *in vacuo*. The crude product was then purified using column chromatography (mobile phase; hexane: ethyl acetate 3:1).

Physical Properties: Yield: 37%. mp: 89–92 °C. ^1^H NMR (400 MHz, CDCl_3_): *δ* 7.39 (s, 1H), 7.31–7.22 (m, 6H), 6.78–6.59 (m, 2H), 4.17 (t, 2H, *J* = 7.0), 3.10–3.06 (t, 2H, *J* = 7.1), 2.13 (s, 3H). ^13^C NMR (100 MHz, CDCl_3_): 162.6, 160.9, 154.8, 139.3, 137.7, 128.9, 128.6, 127.8, 126.7, 122.1, 113.2, 112.7, 101.2, 69.1, 35.5, 16.9. HR-ESI [M + H] ^+^: calc. 281.1178, exp. 281.1171. FT–IR (ATR): *ν*_max_ (cm^−1^) 3026, 2850, 1612, 1284, 1247.

#### 2.1.4. Synthesis of 3-methyl-2-oxo-2H-1-benzopyran-7-yl-diethylcarbamate (3)

A microwave compatible glass-vessel was charged with 250 mg (1.4 mmol) of 7-hydroxy-3-methyl-2H-1-benzopyran-2-one (**1**), 295 mg (2.1 mmol) of K_2_CO_3,_ and a catalytic amount of TBAHSO_4_ and dissolved in 10 ml of acetonitrile. Following this, 385 mg (2.8 mmol) of diethyl carbamoyl chloride was added dropwise and stirred at 100 °C for 2.5 h under microwave irradiation (maximum power = 150 W). Once the reaction was complete, K_2_CO_3_ was filtered out and the solvent was removed *in vacuo*. The crude mixture was dissolved in 30 ml ethyl acetate and 15 ml water and transferred to a separatory funnel. The layers were separated and the organic layer was washed with 15 ml of 1 M KOH and then 15 ml of water. The combined aqueous layers were extracted with ethyl acetate (3 × 20 ml). The combined organic layers were then washed with brine and concentrated under reduced pressure. The product was obtained as an amber coloured oil.

Physical Properties: Yield: 65%. mp: wax. ^1^H NMR (400 MHz, DMSO-d_6_): *δ* 7.85 (s, 1H), 7.62–7.60 (d, 1H, *J* = 8.4 Hz), 7.20–7.19 (d, 1H, *J* = 2.1 Hz), 7.11–7.09 (dd, 1H, *J* = 2.2, 8.5 Hz), 3.42–3.37 (m, 2H), 3.36–3.28 (m, 2H), 2.08 (s, 3H), 1.21–1.10 (t, 6H, *J* = 6.6). ^13^C NMR (100 MHz, CDCl_3_): 161.9, 153.5, 153.2, 153.0, 138.7, 127.2, 124.5, 118.3, 116.6, 109.8, 42.3, 41.9, 29.6, 16.9, 14.1, 13.2. HR-ESI [M + H] ^+:^ calc. 276.1236, exp. 276.1235. FT–IR (ATR): *ν*_max_ (cm^−1^) 2920, 1708, 1471, 1316, 1240.

#### 2.1.5. Synthesis of 3-(bromomethyl)-7-(2-phenylethoxy)-2H-1-benzopyran-2-one (4)

A mixture of 114 mg (0.64 mmol) of *N*-bromosuccinimide, 150 mg (0.54 mmol) of 3-methyl-7-(2-phenylethoxy)-2H-1-benzopyran-2-one (**2**) and 33 mg (0.14 mmol) of benzoyl peroxide (75%) were dissolved in 7 ml CCl_4_. The reaction vessel was stirred at room temperature for 7 h and once the reaction was complete the succinimide residue was filtered out. The solvent was removed *in vacuo* and the crude product was purified via flash column chromatography (mobile Phase; hexane:ethyl acetate:chloroform, 4:4:1). An orange solid of compound **4** was obtained.

Physical Properties: Yield: 43%. mp: 101–105 °C. ^1^H NMR (400 MHz, CDCl_3_): *δ* 7.70 (s, 1H), 7.43–7.27 (m, 6H), 6.86–6.83 (dd, 1H, *J =* 2.3, 8.58 Hz), 6.81–6.80 (d, 1H, *J* = 2.1 Hz), 4.41 (s, 2H), 4.25–4.21 (t, 2H, *J* = 7.0), 3.14–3.11 (t, 2H, *J* = 7.1). ^13^C NMR (100 MHz, CDCl_3_): 142.2, 129.0, 128.9, 128.9, 128.6, 128.6, 127.8, 126.8, 121.7, 113.4. 112.7, 101.3, 101.2, 69.3, 69.1, 35.5, 35.5, 28.2. HR-ESI [M + H]^+^: calc. 359.0283, exp. 359.0298. FT–IR (ATR): *ν*_max_ (cm^−1^) 3029, 2918, 1707 1158, 698.

#### 2.1.6. Synthesis of 3-(bromomethyl)-2-oxo-2H-1-benzopyran-7-yl-diethylcarbamate (5)

N-Bromosuccinimide (323 mg, 1.81 mmol) was added to a suspension of 3-methyl-2-oxo-2H-1-benzopyran-7-yl-diethylcarbamate (250 mg, 0.91 mmol) (**3**) in 9 ml CCl_4_. Following this benzoyl peroxide (75%) (192 mg, 0.80 mmol) was added to the mixture. After heating at reflux for 15 h, the hot reaction mixture was filtered to remove the succinimide by-product. The solvent was removed *in vacuo* and the crude product was purified via flash column chromatography (Mobile Phase; DCM: hexane: ethyl acetate, 4:3:1) to obtain a white solid.

Physical properties: Yield: 15%. mp: 84–88 °C. ^1^H NMR (400 MHz, CDCl_3_): *δ* 7.83 (s, 1H), 7.48–7.46 (d, 1H, *J* = 8.6 Hz), 7.15 (d, 1H, *J* = 1.9 Hz), 7.13–7.10 (dd, 1H, *J* = 2.1, 8.5 Hz), 4.42 (s, 2H), 3.45–3.38 (m, 4H), 1.28–1.19 (m, 6H). ^13^C NMR (100 MHz, CDCl_3_): 154.6, 154.4, 141.6, 128.5, 124.3, 118.9, 116.0, 110.2, 42.4, 42.1, 40.6, 35.1, 28.9, 27.7, 14.3, 13.3. HR-ESI [M + H]^+^: calc. 354.0341, exp. 354.0344. FT–IR(ATR): *ν*_max_ (cm^−1^) 2979, 2920, 1615, 1246, 1147.

#### 2.1.7. Synthesis of 7-(2-phenylethoxy)-3-{[(prop-2-yn-1-yl) amino] methyl}-2H-1-benzopyran-2-one (6)

A mixture of 115 mg (0.32 mmol) of 3-(bromomethyl)-7-(2-phenylethoxy)-2H-1-benzopyran-2-one (5) and 441 mg (3.2 mmol) of K_2_CO_3_ was dissolved in 5 ml dried THF. Propargylamine (44 mg, 0.8 mmol) was added dropwise and the mixture was stirred at room temperature for 48 h. The K_2_CO_3_ was filtered off and the solvent was removed under reduced pressure. The resulting crude product was purified using column chromatography (Mobile phase; hexane: ethyl acetate 2:1) and the product was obtained as a waxy amber solid.

Physical Properties: Yield: 30%. mp: wax. ^1^H NMR (400 MHz, CDCl_3_): *δ* 7.70 (t, 1H, J = 11.8 Hz), 7.36–7.27 (m, 6H), 6.84–6.81 (dd, 1H, *J* = 2.4, 8.4 Hz), 6.80–6.78 (d, 1H, *J* = 2.2H), 4.23– 4.20 (t, 2H, *J* = 6.9 Hz), 3.82 (s, 2H), 3.52 (d, 2H, *J* = 2.4), 3.14–3.1 (t, 2H, *J* = 7 Hz), 2.28 (t, 1H, *J* = 2.4 Hz). ^13^C NMR (100 MHz, CDCl_3_): 161.8, 161.7, 155.1, 140.5, 137.6, 128.9, 128.7, 128.6, 126.7, 122.2, 113.1, 112.7, 101.2, 80.8, 72.5, 69.2, 47.7, 3.4, 35.5. HR-ESI [M + H]^+^: calc. 334.1443, exp. 334.1450. FT–IR(ATR): *ν*_max_ (cm^−1^) 3284, 3027, 2922, 2853, 1706, 1237.

#### 2.1.8. Synthesis of 2-oxo-3-{[(prop-2-yn-1-yl) amino] methyl}-2H-1-benzopyran-7-yl-diethylcarbamate (7)

3-(Bromomethyl)-2-oxo-2H-1-benzopyran-7-yl-diethylcarbamate (**4**) (120 mg, 0.34 mmol) was dissolved in 2.5 ml of THF before adding 480 mg (3.4 mmol) of K_2_CO_3_ and 38 mg (0.68 mmol) of propargylamine. The mixture was stirred at room temperature for 48 h following which the inorganic residue was filtered off after washing with THF. The resulting solution was concentrated *in vacuo* and purified using column chromatography (Mobile phase; DCM: hexane: ethyl acetate in a 20:1:1 ratio) and an off-white solid was obtained.

Physical Properties: Yield: 21%. mp: 124–126° C. ^1^H NMR (400 MHz, CDCl_3_): *δ* 7.72 (s, 1H), 7.46–7.44 (m, 1H), 7.13 (d, 1H, *J* = 2.0 Hz), 7.10–7.07 (dd, 1H, *J* = 2.2, 8.4 Hz), 3.83 (s, 2H), 3.50 (s, 2H), 3.45–3.38 (m, 4H), 2.26–2.25 (t, 1H, *J* = 2.1 Hz), 1.28–1.19 (m, 8H). ^13^C NMR (100 MHz, CDCl_3_): 161.2, 153.9, 153.8, 153.2, 140.7, 128.4, 124.1, 118.7, 116.5, 109.9, 77.8, 74.5, 52.5, 42.9, 42.4, 42.0, 29.7, 14.3, 13.3. HR-ESI [M + H]^+^: calc. 329.1496, exp. 329.1495. FT–IR(ATR): *ν*_max_ (cm^−1^) 2921, 2851, 1634, 1616, 1245.

### 2.2. Biological studies

#### 2.2.1. Cholinesterases assay

Cholinesterase activity is determined using a modified colorimetric method first described by Ellman^40^. The test compounds and positive control (donepezil) were dissolved in DMSO to prepare 10 mM stock solutions. The stock was then further diluted by factors of ten to produce solutions of 1 mM, 100 µM, 10 µM, 1 µM, and 0.1 µM which corresponds to concentrations of 100 µM, 10 µM, 1 µM, 0.1 µM, and 0.01 µM in the final reaction mixture. The test concentrations were stored in the refrigerator until the day of the assay. Trisma HCl buffer (50 mM, adjusted to pH 8 with 2 N NaOH) was prepared as the buffer and used to prepare enzyme stock solution (22 U/ml for *Electrophorus electricus* AChE and 12 U/ml BuChE from equine serum), 15 mM acetylthiocholine iodide, 15 mM S-butyrylthiocholine iodide and 1.5 mM DTNB (Ellman’s reagent). The enzyme stock solution was stabilised with 1% bovine serum albumin and stored in aliquots at −80 °C. A clear, flat bottom 96 well plate was used to perform the experiments with each concentration performed in triplicate. Prior to the assay the background absorbance for each compound at the different test concentrations was measured and accounted for when reading the final absorbance. Before performing the assay, the enzyme was diluted to 0.88 U/ml for AChE and 0.48 U/ml for BuChE. In each well 148 µl DTNB, 50 µl of the enzyme and 2 µl of the test compound or control (donepezil in the positive control and DMSO in the negative control well) were added and incubated at 25 °C for 10 min. The concentration of DMSO was kept constant at less than 1% in order not to influence the assay results. Following this, 30 µl of the substrate was added to each well simultaneously using a multi pipette. The plate was then placed in a Rayto^®^ RT-2100C microplate reader and the absorbances were measured at 405 nm every 60 s over a 20-min period. The data obtained was used to calculate the maximum inhibition over the 20-min period and this was used to plot a dose-response graph, from which the IC_50_ values of the compounds could be extrapolated. A one-sample *t*-test was performed on the data to test for statistical significance.

#### 2.2.2. MAO assays

The test compounds and positive control (clorgiline for MAO-A and selegiline for MAO-B) were dissolved in DMSO to prepare 10 mM stock solutions. The stock was then further diluted by factors of ten to produce solutions of 1 mM, 100 µM, 10 µM, 1 µM, and 0.1 µM which would correspond to concentrations of 100 µM, 10 µM, 1 µM, 0.1 µM, and 0.01 µM in the final reaction mixture. A potassium phosphate buffer (KH_2_PO_3_ 100 mM, pH 7.4, 0.9% w/v NaCl) was prepared and refrigerated until further use. The enzyme stock solutions were prepared by dissolving 2.5 mg of enzyme in 33.33 ml of phosphate buffer to produce stock solutions of 0.075 mg/ml which were stored in aliquots at −80 °C until use. Once in the final reaction mixture, this enzyme solution will have a concentration of 0.0075 mg/ml. The substrate was prepared by dissolving kynuramine in potassium buffer and was prepared as two separate concentrations; 750 µM for MAO-A and 500 hx00B5;M for MAO-B. When placed in the reaction mixture, these solutions ended up with final concentrations of 45 µM for MAO-A and 30 µM for MAO-B respectively. The fresh substrate was prepared on the day of the assay. The assay was carried out in 2 ml Eppendorf vials. To each vial, 207.5 hx00B5;l of phosphate buffer was added followed by 2.5 hx00B5;l of the respective test compound or control (positive control: clorgiline for MAO-A or rasagiline for MAO-B; control: DMSO). Following this, 25 µl of the enzyme stock were added to each vial in 10 s intervals and the vials were incubated at 37 °C for 10 min. After this time period 15 µl of kynuramine (750 µM for MAO-A and 500 µM for MAO-B) was added at 10 s intervals and further incubated for 20 min. To stop the reaction at the end of this time 150 hx00B5;l of 2 N NaOH was added and the mixture was shaken. Using a micropipette 80 hx00B5;l of the mixture was transferred to a well in a black 96 well plate. The plate was placed in the fluorescent plate reader and read at an excitation/emission wavelength of 310 nm/410 nm. Each assay was run in triplicate and the data were analysed with Graph Pad Prism 8 software (San Diego, USA).

#### 2.2.3. MAO-B reversibility study

To investigate the reversibility of the observed MAO enzyme inhibition, time-dependent inhibition studies were carried out on compounds **6** and **7**. Briefly, the recombinant human MAO-B (0.0075 mg/ml) enzyme was preincubated with either **6** or **7** for periods of 0, 15, 30, 60 min at 37 °C. The concentration of inhibitor in these incubations was approximately twofold the measured IC_50_ value for the inhibition of the MAO-B enzyme. Potassium phosphate buffer (100 mM, pH 7.4, made isotonic with NaCl) was used as the incubation medium. A final concentration of 30 hx00B5;M kynuramine for MAO-B was then added to the preincubated enzyme preparations and the resulting 250 µl reactions were incubated at 37 °C for 15 min. The reactions were terminated with the addition of 200 µl NaOH (2 N) and a volume of 600 µl distilled water was added to each reaction. The fluorescence of the MAO-B generated 4-hydroxyquinoline in the supernatant fractions were measured using a fluorescent microplate reader at an excitation wavelength of 310 nm and an emission wavelength of 400 nm. Time-dependent graphs were plotted against the relative fluorescent unit readings (RFU).

#### 2.2.4. Cell line and culture conditions

The human neuroblastoma SH-SY5Y cells were generously donated by collaborators from the Blackburn Laboratory, University of Cape Town. Cells were cultured in monolayer in Dulbecco’s Modified Eagle Medium (DMEM, Gibco, Life Technologies Corporation UK), supplemented with 10% foetal bovine serum (FBS, Gibco, Life Technologies Corporation, Paisley, UK), 100 U/ml penicillin, and 100 µg/ml streptomycin (Lonza Group Ltd., Verviers Belgium). Cultures were incubated at 37 °C in a humidified atmosphere with 5% CO_2_. The growth medium was replaced every 3 days and cells were sub-cultured when they attained 70 to 80% confluency using a solution of 0.25% trypsin EDTA (Lonza Group Ltd., Verviers, Belgium).

#### 2.2.5. MTT assay

SH-SY5Y cells were plated in growth medium in the flat bottom 96 well plates at a density of approximately 7500 cells/well. The cells were allowed to adhere to the plate surface for 24 h and following this their media were replenished with fresh media containing the test compounds at 10 µM, 50 µM and 100 µM concentrations dissolved in DMSO. Vehicle control cells were treated with DMSO, at a concentration similar to that of the highest concentration of the test compounds. Following an incubation period of 48 h, 10 µl of MTT solution (5 mg/ml) was added to each well. This was further incubated for 4 h and the formazan formed was solubilised with 100 µl of DMSO. The plates were read using a BMG Labtech Omega^®^ POLARStar plate reader spectrophotometer (Ortenberg, Germany) to determine the absorbance recorded at a wavelength of 570 nm. The percentage cell viability was calculated relative to the vehicle control using the following formula:

$$\text{Cell}\text{viability}\%=\left(\frac{\text{Absorbance}\text{of}\text{treated}\text{well}}{\text{Absorbance}\text{of}\text{untreated}\text{well}}\right)\times 100$$





#### 2.2.6. Neuroprotection studies

##### 2.2.6.1. MPP^+^ induced neurotoxicity

SH-SY5Y cells were seeded onto a 96-well plate in a growth medium at a density of 10,000 cells/well. Cells were pre-treated with test compounds at a concentration of 10 µM and thereafter 2000 µM MPP^+^ following a 2-h incubation period. Afterwards, cells were incubated for 24 h and MTT colorimetric assay was used to measure cell viability relative to untreated control. To determine the data’s statistical significance, the One-way ANOVA Turkey’s multiple comparison test was conducted on all experimental replicates using GraphPad Prism^®^ 8 (San Diego, USA).

##### 2.2.6.2. Amyloid-beta 25–35 induced neurotoxicity

Human neuroblastoma cells (SH-SY5Y) were grown in a culture medium (DMEM/F-12) and supplemented with 10% foetal bovine serum. Prophylactically, 1% v/v of penicillin/streptomycin was added to the medium. Cells were maintained by incubation at 37 °C with 5% carbon dioxide. All experiments were performed by seeding the cells into 24-well plates. Once confluent, the cells were then pre-treated with compounds **6** and **7** (10 µM) for 1 h, followed by Aβ 25–35 addition (20 µM) and incubated for 24 h. The untreated control cells were treated with DMEM which contained DMSO and were incubated in the same manner as the treated cells. After incubation for the set period of time the MTT colorimetric assay, as discussed under 2.2.5, was used to measure cell viability relative to untreated control. The level of significance was analysed using One-way ANOVA statistical analysis and expressed using the *p*-value.

### 2.3. Molecular modelling

#### 2.3.1. AChE docking studies

Molecular Operating Environment (MOE) 2018 on a Windows platform was used for the docking studies. The crystal structure of the AChE enzyme was obtained from the PSLIO/PDB data bank co-crystallised with donepezil (Code: 1EVE). Prior to the studies the enzyme was first checked for missing atoms and bonds. Partial charges, ionisation state and hydrogens were added to the macromolecular structure using the 3D protonation function in the MOE application. The co-crystallised ligand was then selected to identify which binding site to use as the pocket for docking. The prepared enzyme was then saved in this state for use in the docking studies. The test ligands were drawn using ChemSketch 2016 software, saved as MDL files (V3000) and prepared for docking by energy minimising them in MOE using the MMFF94 force field. A database of these compounds was created and docking performed. Poses were generated using Triangle Matcher placement method and rescored using the ASE scoring function. The best poses were visually inspected and binding interactions analysed. To confirm the accuracy of the protocol it was repeated three times and generated RMSD values <2 Å from the co-crystallised ligand. This showed that the protocol could correctly predict the binding orientations.

#### 2.3.2. MAO docking studies

Docking of the ligands was performed with MOE 2018. The co-crystallised MAO enzymes were obtained from the PSLIO/PDB data bank with MAO-A co-crystallised with clorgiline (Code: 2BXS) and MAO-B co-crystallised with safinamide (Code: 2V5Z). Prior to the studies, the enzyme was first checked for missing atoms and bonds. Partial charges, ionisation state, and hydrogens were added to the macromolecular structure using the 3D protonation function in the application. The co-crystallised ligand was then selected to identify which binding site to use as the pocket for docking. The prepared enzyme was then saved in this state for use in docking studies. The test ligands were drawn using the ChemSketch 2016 software, saved as MDL files (V3000) and prepared for docking by energy minimising them in MOE using the MMFF94 force field. A database of these compounds was created and docking performed. Poses were generated using the Triangle Matcher placement method and rescored using the ASE scoring function. The best poses were visually inspected and binding interactions analysed. To confirm the accuracy of the protocol it was repeated three times and generated RMSD values < 1.5 Å from the co-crystallised ligand. This showed that the protocol could correctly predict the binding orientations.

### 2.4. *In silico* pharmacokinetic and drug-likeness evaluation

To evaluate the drug-likeness and pharmacokinetic profiles of compounds **6** and **7**, the SwissADME web tool (http://www.swissadme.ch/) was utilised. The structures were drawn using ChemSketch (2016) and saved as MDL molfiles. The files were converted to SMILES (simplified molecular-input line-entry system) format which were then input into the web tool and the pharmacokinetic parameters were generated.

## 3. Results and discussion

### 3.1. Chemistry

**Scheme 1.**
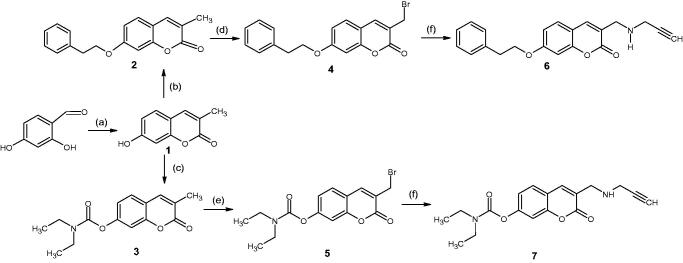
Synthesis pathway of the test compounds. Reagents and conditions: (a) Sodium propionate, propionic anhydride, piperidine, reflux, 6 h; (b) 2-bromoethylbenzene, NaH, acetonitrile, MW @ 150 W, 80 °C, 5 h; (c) diethyl carbamoyl chloride, K_2_CO_3_, acetonitrile, MW @ 150 W, 80 °C, 2.5 h; **(d)** *N*-bromosuccinimide, benzoyl peroxide, CCl_4_, stir at room temperature, 7 h; (e) *N*-bromosuccinimide, benzoyl peroxide, CCl_4_, reflux, 15 h; (f) propargylamine, K_2_CO_3_, dry THF, stir for 48 h.

The synthesis of the compounds (Scheme 1) commenced with a Pechmann condensation of dihydroxybenzaldehyde to obtain 7-hydroxy-3-methyl-coumarin (**1**) which served as the base scaffold. This scaffold was chosen due to the ease of substitution that the –OH group at position 7 allows. Simultaneously, the poor reactivity of the 3-CH_3_ group protects the scaffold from unwanted substitution reactions occurring during subsequent reactions. Compound **1** was then functionalised at position 7 via either an etherification reaction with 2-bromoethylbenzene (**2**) or carbamate formation with diethyl carbamate (**3**). The synthesis of both **2** and **3** was optimised with the use of microwave (MW) irradiation to assist these S_N_2 reactions, following the failure of traditional synthetic methods (such as reflux) which took longer and produced inferior yields (Table 1). The 7-substituted compounds were then α-brominated with *N*-bromosuccinimide to produce a -CH_2_Br group (compounds **4** and **5**) at position 3, which further facilitated the substitution of a propargylamine moiety to produce either compound **6** or **7**.

**Table 1.** Optimisation of reaction conditions using microwave-assisted methods for compounds **2** and **3.**

Compound	Conditions	Yield^a^
2	Ethanol, NaH	0%
	Stir at room temp., 11 h	
	Acetonitrile, NaH	37%
	MW @ 150 W, 80 °C, 5 h	
3	Dimethoxyethane, K_2_CO_3_	0%
	Stir at room temp., overnight	
	Dimethoxyethane, K_2_CO_3_	9%
	Reflux, 12 h	
	Acetonitrile, K_2_CO_3_	27%
	MW @ 150 W, 100 °C, 6 h	
	Acetonitrile, K_2_CO_3_, TBAHSO_4_	65%
	MW @ 150 W, 100 °C, 2.5 h	

^a^Percentage yield was calculated from the amount of 7-hydroxy-3-methyl-coumarin (**1**) used.

All of the synthesised compounds were characterised using routine spectroscopic methods (NMR, IR, and MS). The ^1^H NMR spectra of all the synthesised compounds presented two singlets which were paired with a set of two doublets between 7.1 ppm and 7.8 ppm. This set of peaks is characteristic of the four aromatic protons found in the 3,7-substituted coumarin nucleus and was largely conserved across all the compounds. The –N(CH_2_CH_3_)_2_ chain present in the 7-carbamate derivatives (**3**, **5**, and **7**) appeared as a pair of multiplets at 3.3–3.5 ppm and 1.20–1.28 ppm. These multiplets are representative of the chemically equivalent –CH_2_ and –CH_3_ groups on this alkyl chain, respectively. Likewise, the spectra for the 7-phenylethyloxy derivatives (**2**, **4**, and **6**) had a characteristic multiplet representing the five hydrogens of the phenyl moiety, which appeared in the aromatic region. The alkyl chain linking this phenyl ring to the coumarin nucleus was represented by two sets of triplets between 3.1 ppm and 4.2 ppm. This relative downfield shift is due to the deshielding effect caused by the oxygen atom in the ether linkage with the coumarin nucleus. The presence of a 3-propargyl moiety in compounds **6** and **7** was confirmed by the disappearance of the singlet representing 3-CH_2_Br present in the spectra of precursor compounds **4** and **5**. In its place, a doublet (for –CH_2_) and triplet (for CH) appeared. ^13^C NMR data were used to confirm the number of carbons in each structure and their respective environments corresponded with what was expected for the structures. The FT-IR data were used to qualitatively identify the functional groups present and compounds with similar substitutions had spectra with similar signal patterns. HR-MS data confirmed the molecular mass as well as molecular formulae of the compounds.

### 3.2. Biological evaluations

#### 3.2.1. MAO inhibition studies

The MAO inhibitory activity of the compounds was assessed using a fluorometric assay with recombinant human MAO-A and human MAO-B enzymes. The compounds demonstrated good inhibition of both MAO isoforms (Table 2). Against MAO-A, compounds **2**, **4**, and **5** performed the best, displaying IC_50_ values between 0.36 and 0.66 µM. Compounds **4** and **5** demonstrate that replacing the 3-CH_3_ with 3-CH_2_Br produced a significant increase in activity of the compounds, with the lowest IC_50_ values of 0.48 µM and 0.36 µM for **4** and **5**, respectively. Subsequent replacement of 3-CH_2_Br with the 3-propargylamine moiety led to a decrease in activity towards MAO-A of several magnitudes as seen with compounds **6** and **7**.

**Table 2.** *In vitro* biological activities of the synthesised and reference compounds.

Compound	AChE IC_50_ (µM)	BuChE IC_50_ (µM)	MAO-A IC_50_ (µM)	MAO-B IC_50_ (µM)	SI MAO-B^a^ (µM)	MPP^+^ neuroprotection^b^ (10 µM)
**Donepezil**	0.007	4.40	n.d.	n.d.	n.d.	n.d.
**Clorgiline**	n.d.	n.d.	0.001	n.d.	n.d.	n.d.
**Selegiline**	n.d.	n.d.	n.d.	0.010	n.d.	n.d.
**1**	>100	20.25	33.40	16.36	2.04	26.24%
**2**	>100	>100	0.658	0.014	47.7	24.85%
**3**	>100	>100	2.822	0.498	5.67	15.59%
**4**	>100	>100	0.476	>100	n.d.	22.11%
**5**	108	23.27	0.355	0.333	1.05	21.86%
**6**	>100	>100	3.86	0.029	133.2	35.96%
**7**	>100	>100	20.80	0.101	205.9	28.19%

^a^MAO-B selectivity index = IC_50_(MAO-A)/IC_50_(MAO-B). ^b^Percentage neuroprotection values calculated as the difference between the final percentage cell viability of the test compound treated cell line and that of the MPP^+^ only treated cell line. n.d. = not determined

Comparatively, the compounds performed significantly better against MAO-B with five of the test compounds displaying nanomolar IC_50_ values (Table 2). Compounds **2** and **6** performed best in this assay with potent IC_50_ values of 14 nM and 29 nM, respectively. The calculated selectivity index (SI) showed that the compounds exhibit selectivity towards MAO-B, with the exception of compound **4**. This is quite important as this is the isoform found predominantly in the brain and avoids the potential of a tyramine-induced hypertensive crisis developing as seen with MAO-A inhibitors.

The results confirm that the initial substitutions to position 7 increased both the inhibitory capacity and MAO-B selectivity of the coumarin scaffold. The phenylethyloxy moiety conferred superior inhibition and selectivity when compared to the carbamate moiety in this regard (see Table 2). Incorporation of the progylamine moiety appears to not only increase the derivatives potency towards MAO-B, but also has the greatest influence on the compounds’ selectivity. The 3-propargylamine derivatives **6** and **7** displayed between 130 and 206 times greater selectivity to MAO-B when compared to MAO-A. Together, these results show that whereas, compounds **2** was more active in the inhibition of MAO-B, compounds **6** and **7** showed better selectivity and this can be attributed to the addition of the propargylamine moiety.

#### 3.2.2. MAO molecular modelling studies

##### 3.2.2.1. MAO-A docking studies

**Figure 3.**
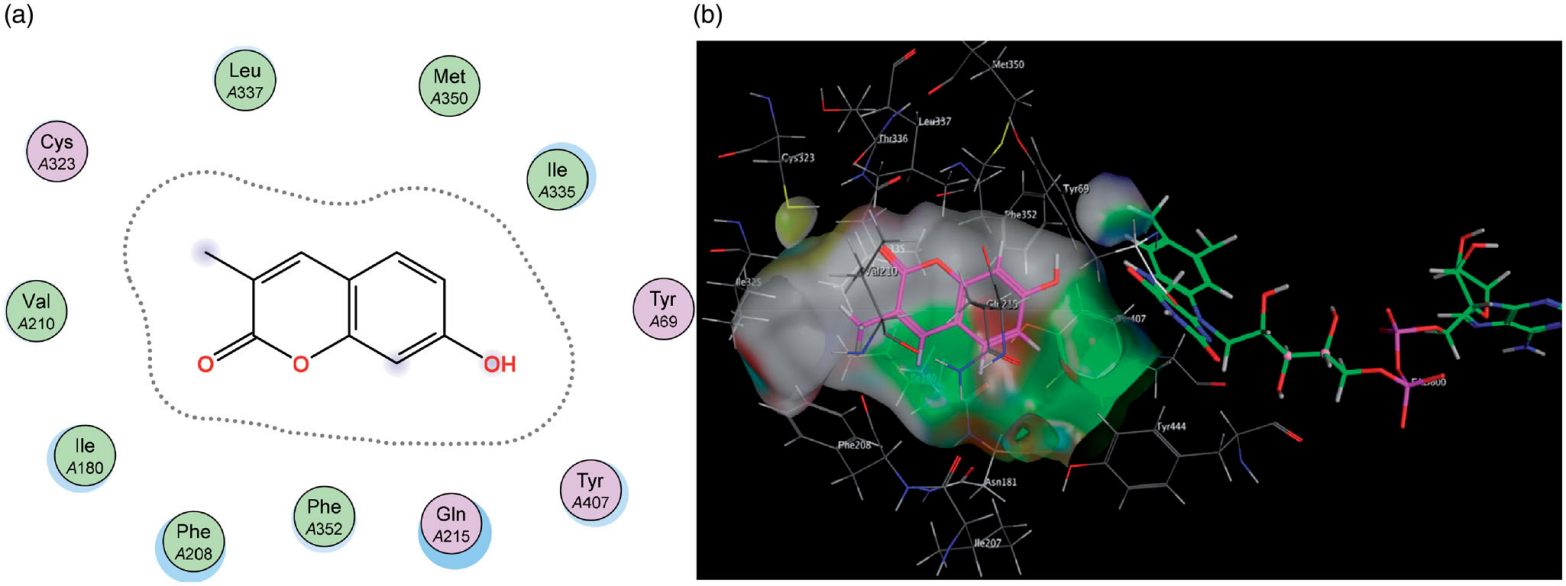
(a) Diagrammatic representation of compound **1**’s lack of binding interactions with amino acids in MAO-A’s active site. (b) 3D docking simulation showing **1** indicated in pink within the MAO-A active site.

**Figure 4.**
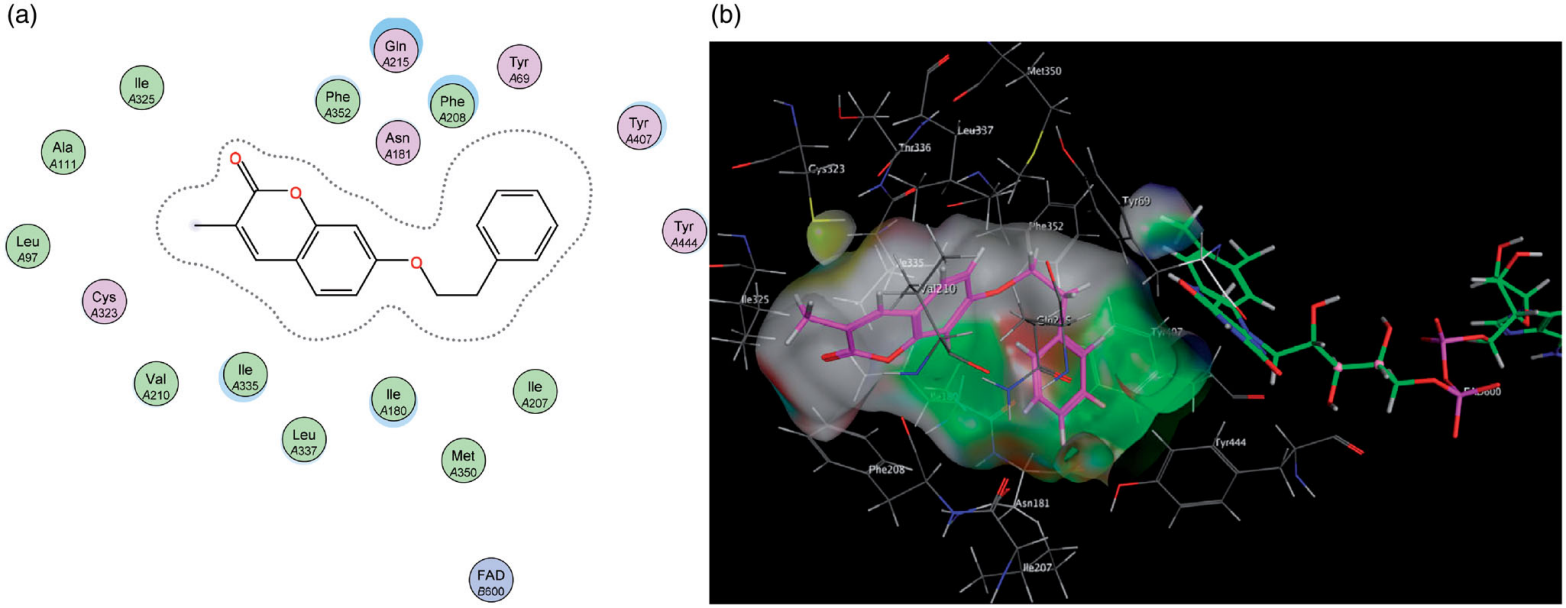
(a) 2D representation showing the 7-phenylethyl derivative **2**’s orientation in MAO-A’s substrate cavity. (b) 3D docking simulation showing **2** indicated in pink within the MAO-A active site.

Molecular Operating Environment (MOE) was used to perform docking studies with the test compounds in an attempt to shed light on the preliminary structure-activity relationships responsible for the results obtained in the assay. When the base scaffold, compound **1**, was docked within the MAO-A active site it was observed that the coumarin nucleus simply enters the entrance cavity, with no significant binding interactions taking place (Figure 3). This lack of binding correlates to the compound’s low activity observed in the assay. The docking simulations also confirmed that the superior activity of the 7-phenylethyloxy derivatives arose from the phenylethyloxy moiety allowing compounds **2** and **4** to assume orientations that occupy MAO-A’s rounder-shaped substrate cavity better than the other compounds (Figure 4). By contrast, the 7-carbamate derivatives **3** and **5** are seen to only exhibit weak sidechain interactions with the Cys 323 residue (near the entrance of the catalytic site) and the oxygen atom on the carbamate moiety.

**Figure 5.**
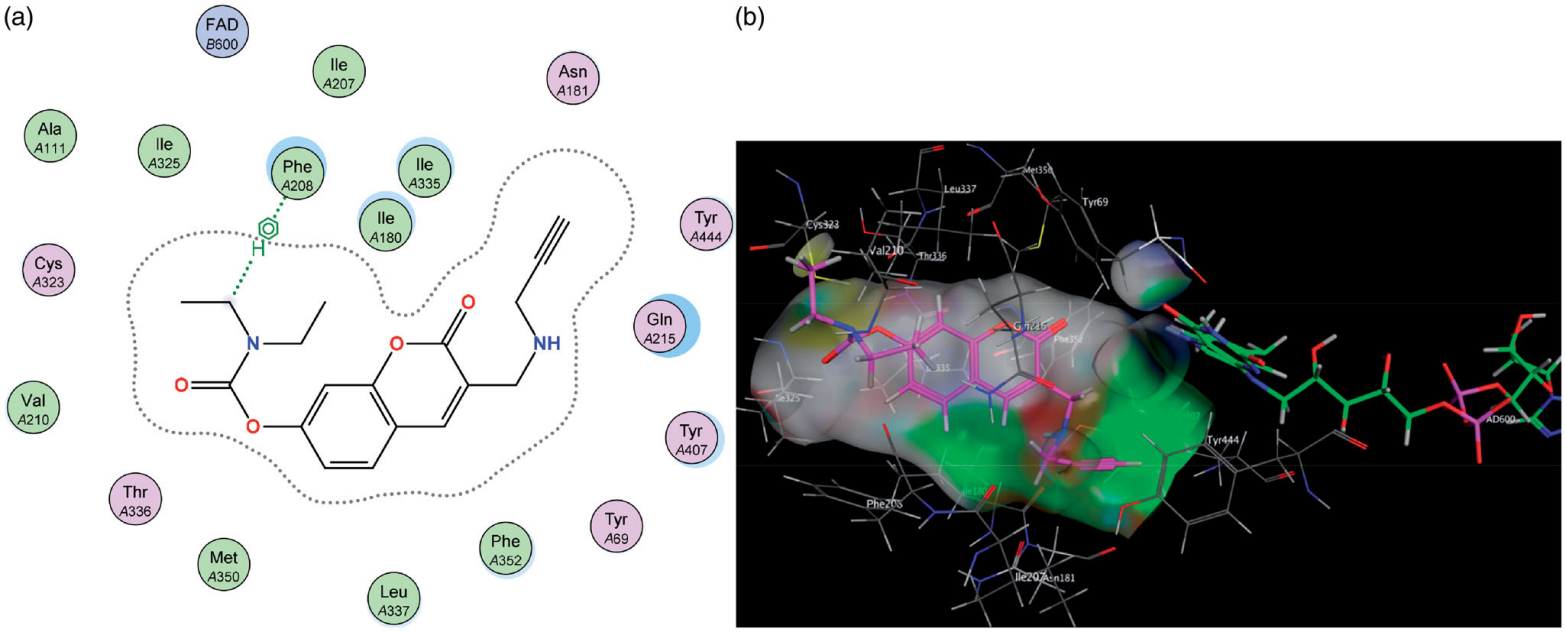
(a) 2D interaction map showing compound **7**’s orientation and binding in MAO-A’s substrate cavity. (b) 3D docking simulation showing **2** indicated in pink within the MAO-A active site.

Figure 5 shows that the addition of the propargylamine scaffold produced molecules that are unable to fit into MAO-As active site. Docking conformations of compounds **6** and **7** demonstrate that the molecules lack the flexibility to fit and subsequently interact with amino acids in the cavity’s active site. This correlates with the relatively poor MAO-A inhibitory capacity of these compounds reported in the assays (Table 2).

##### 3.2.2.2. MAO-B docking studies

**Figure 6.**
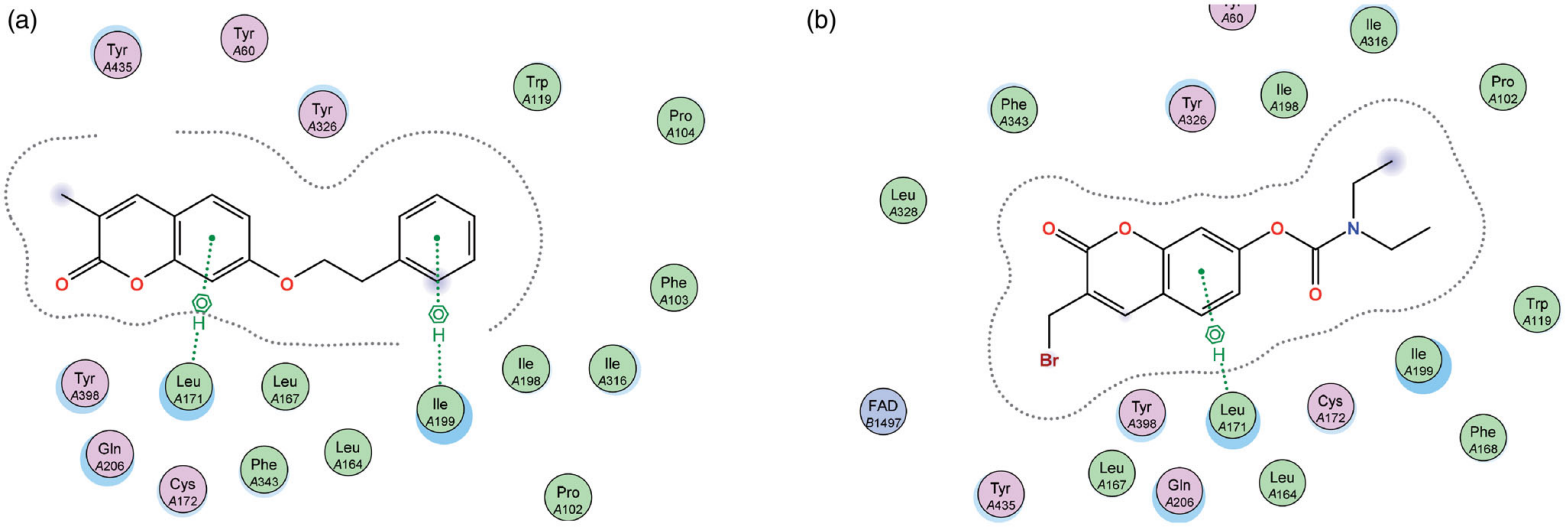
Diagrammatic representation showing binding interactions of the (a) 7-phenylethyloxy compounds (represented by **2)** and (b) 7-carbamate compounds (represented by **5**) when docked with MAO-B. This compound’s lack of π–H interaction with Ile 199 may explain its lower activity.

Contrary to MAO-A, for MAO-B it can be observed that the coumarin nucleus **1** fits well into the entrance cavity, forming relatively stable π–H bonds between the aromatic ring and the amino acids Ile 199 and Leu 171 (Figure 6). This hydrogen bond with the Leu 171 residue on the fringe of the substrate cavity was observed and conserved for the rest of the compounds and may explain their selectivity towards MAO-B. The 7-phenylethyloxy derivatives’ (particularly compound **2**) good activity and selectivity can be attributed to the previously noted interactions with Leu 171 coupled with new stable π–H bonds which form between the phenylethyl moiety and the Ile 199 residue. This interaction is important as the Ile 199 residue is critical in forming part of the gating mechanism which allows access to MAO-B’s substrate cavity^41^. The 7-carbamate compounds retain the π–H interaction with Leu 171 which was first observed with **1**, however, they lack the hydrogen bond with Ile 199 and this may account for their lower activity compared to their 7-phenylethyloxy counterparts.

**Figure 7.**
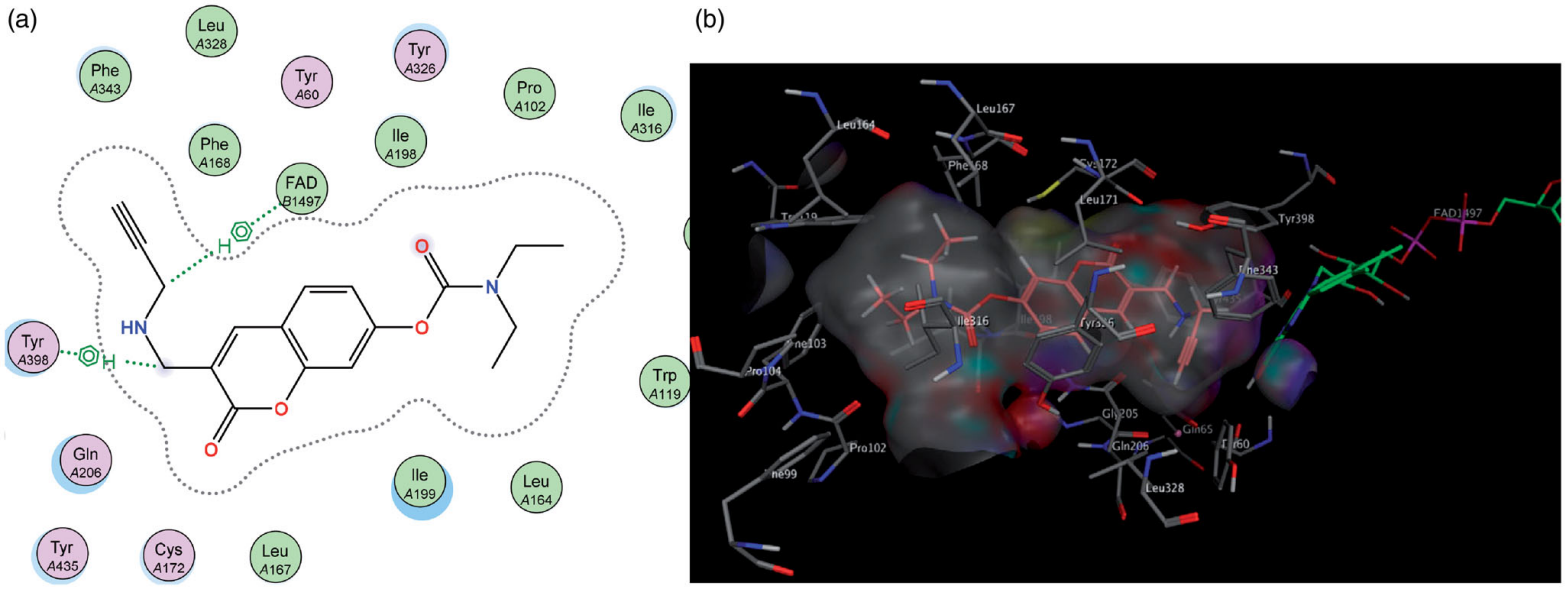
(a) 2D interaction map showing compound **7**’s orientation and π–H binding with FAD and Tyr 398 in MAO-B’s substrate cavity. (b) 3D docking simulation showing **7** indicated in pink within the MAO-B active site.

Similarly, in contrast to MAO-A, the elongated nature of the MAO-B active site allows for better accommodation of the 3-propargylamine derivatives **6** and **7**. It was observed that both compounds were able to interact with the crucial flavin adenine dinucleotide (FAD) cofactor to varying extents due to the inclusion of the propargylamine moiety. The FAD cofactor is essential for substrate catalysis and thus compounds that can come into close proximity or bind to this cofactor are known to inhibit the enzyme function to a greater extent^42^. Compound **6** maintains the previously mentioned π–H bonding with Cys 172 as well as its propargylamine moiety orienting in close proximity to FAD. Compound **7**’s propargylamine moiety forms crucial π–H bonds with the cofactor. From the low nanomolar IC_50_ and high SI values displayed by these compounds, the importance of the propargylamine moiety can be seen in its ability to produce highly potent and selective inhibitors of MAO-B (Figure 7) .

#### 3.2.3. MAO-B reversibility studies

**Figure 8.**
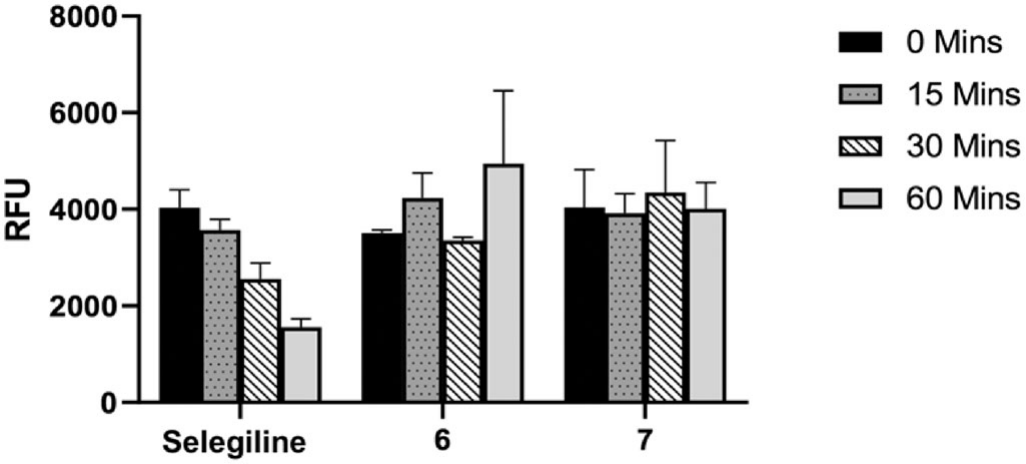
Time-dependent inhibition of the recombinant human MAO-B catalysed oxidation of kynuramine by selegiline, **6** and **7**. The enzyme was preincubated for various periods of time (0–60 min, *x*-axis). Data are expressed as the mean RFU (relative fluorescent units) ± SEM of three independent experiments.

To determine the binding mode of the studied compounds on MAO-B, the time dependency of enzyme inhibition was measured. If the compounds form a covalent or irreversible adduct with the enzyme, a time-dependent reduction of enzyme activity would be expected. In this regard, the time-dependent inhibition of MAO-B by the most promising and selective MAO-B inhibitors namely **6** and **7**, were evaluated (Figure 8). Briefly, recombinant human MAO-B was preincubated with the test compounds for periods of 0, 15, 30, and 60 min prior to starting the enzyme reaction and the residual rates of the MAO-B catalysed oxidation of kynuramine were measured. For this purpose, the concentrations of the test compounds chosen were approximately twofold the measured IC_50_ values for the inhibition of the respective enzymes. Selegiline, an irreversible inhibitor of the MAO-B enzyme with a propargylamine functional group within its structure, was used as a reference compound. As shown in Figure 8, MAO-B enzyme activity was not reduced by the propargylamine containing coumarins **6** and **7** with increased preincubation time. However, selegiline shows a clear time-dependent reduction in activity confirming its irreversible mode of inhibition. This indicates that both compounds **6** and **7** can be considered as reversible inhibitors of MAO-B and that the propargylamine function does not bind to the FAD co-factor covalently as is the case in selegiline^43^^,^^44^. A benefit of reversible MAO inhibitors is that they can possibly reduce the toxicity of the molecules as the effect of the inhibitor can be reversed when the drug is withdrawn^45^^,^^46^.

#### 3.2.4. Cholinesterase inhibition studies

The AChE and BuChE inhibitory capacity of the compounds was tested using a modified method of the assay developed by Ellman^47^. The results of the assay showed that generally, the compounds had low inhibitory activity against AChE up to a 100 µM concentration. The 3-bromoethyl derivative **5** demonstrated the best activity exhibiting 49% enzyme inhibition at the aforementioned concentration and an IC_50_ value of ∼108 µM. The results illustrate that substitution with either the carbamate or phenylethyl moieties at position 7 does not solely confer significant AChE inhibitory activity to the scaffold, as compounds **2** and **3** have similar activity to **1**. However, the α-bromination of the methyl at position 3 to produce -3-CH_2_Br demonstrated a significant increase in the compound’s inhibitory capacity. As observed with compounds **6** and **7**, the 3-propargylamine compounds had diminished activity compared to the 3-bromomethyl of compound **5**. The increased length of these 3-propargylamine derivatives was expected to correlate with an increase in inhibitory activity, however, their activity was only marginally better than the shorter 3-methyl derivatives (**2** and **3**).

Interestingly, the test compounds were more active against BuChE than AChE with higher percentage inhibitions at a 100 µM concentration. Compounds **1** and **5** were the most active inhibitors, with IC_50_ values of 20.25 µM and 23.27 µM respectively. Compound **1**, having the lowest IC_50_ value, demonstrates that neither of the substitutions at the 7-position enhanced the latent BuChE inhibitory capacity of the coumarin scaffold. Compound **5** was the exception having an IC_50_ close to that of **1**, however, there is no significant correlation between the nature of the substitution and the compound’s resultant BuChE inhibitory activity. Based on their higher overall inhibition towards BuChE, the tested compounds can be classified as weakly selective BuChE inhibitors. The compounds’ selectivity may be ascribed to them having stronger binding interactions with the higher density of hydrophobic amino acids lining the active-site gorge of BuChE^40^. The larger volume of BuChE’s active site may also allow the enzyme the ability to accommodate the compounds better than in AChE^48^.

#### 3.2.5. Cholinesterase molecular modelling studies

**Figure 9.**
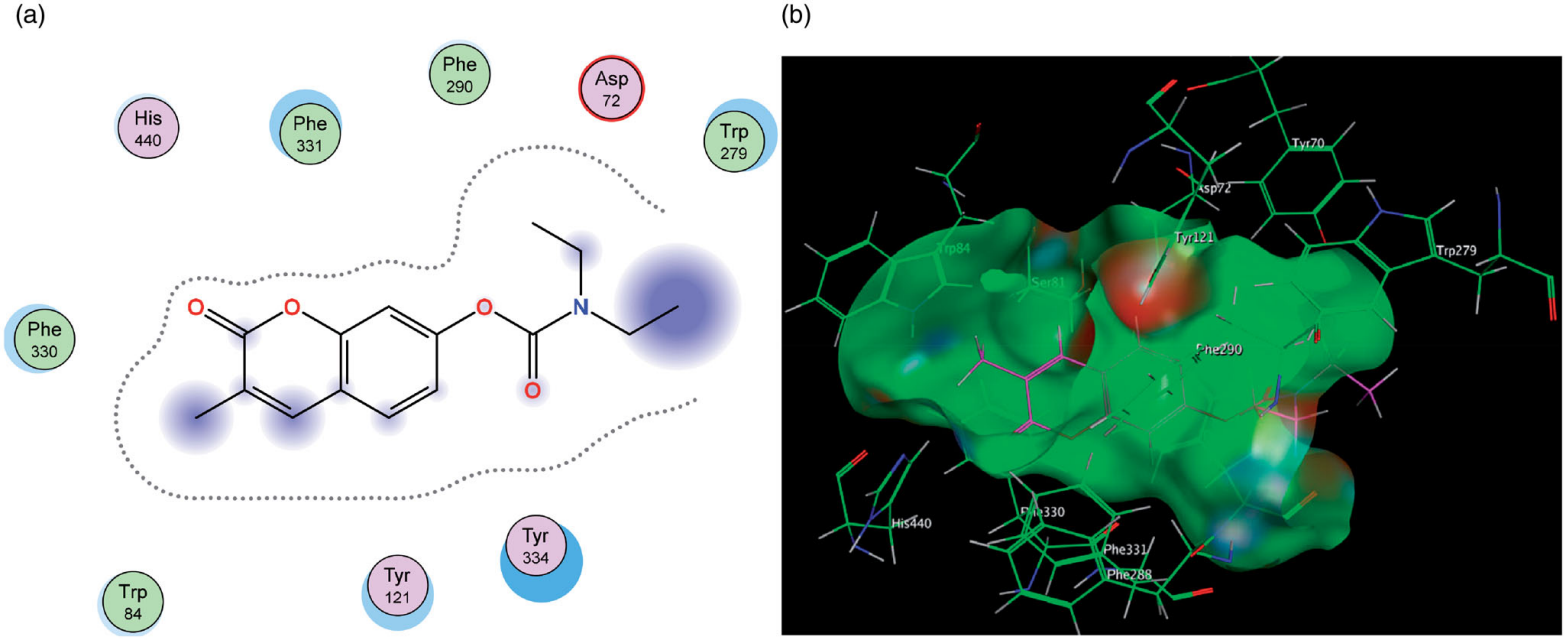
(a) Diagrammatic representation of compound **3**’s lack of binding interactions with AChE. (b) 3-Dimensional representation of docking simulation (bottom) with 3 indicated in pink and AChE’s active site’s amino acids indicated in green. The carbamate portion of the molecule towards the left does not participate in any interactions and lies towards the entrance of the active site.

The AChE enzyme active site is an ellipsoid-shaped gorge, consisting of two main sites termed the peripheral anionic site (PAS) (at the entrance of the active site) and the catalytic binding site (CAS) containing the catalytic triad^49^^,^^50^. The PAS is made up of largely aromatic amino acids such as Trp 279 and is responsible for guiding and stabilising substrates in the active site. The initial predictions from literature anticipated that the coumarin nucleus will sit in the PAS of the enzyme and the various substituents interact with the CAS and catalytic triad^51^^,^^52^. The best-ranked docking results show that instead, the initial substitutions at position 7 performed in compounds **2** and **3** orients the coumarin nucleus away from the PAS and towards the CAS (Figure 9). When the molecules are oriented in this way, neither compound is able to form favourable interactions with amino acids in either of these regions.

**Figure 10.**
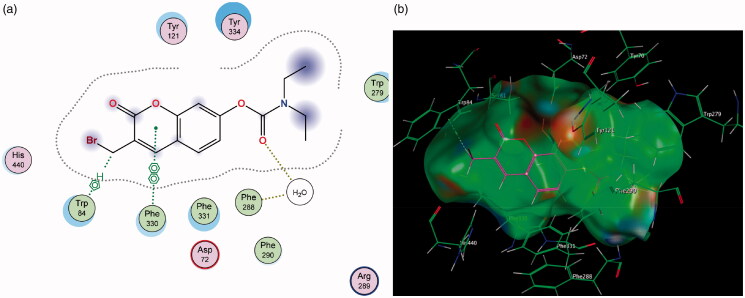
(a) Diagrammatic representation of compound **4**’s binding interactions with Trp 84 and Phe 330 in the CAS of AChE. (b) 3-Dimensional representation of docking simulation (bottom) with **4** indicated in pink and AChE’s active site’s amino acids indicated in green.

**Figure 11.**
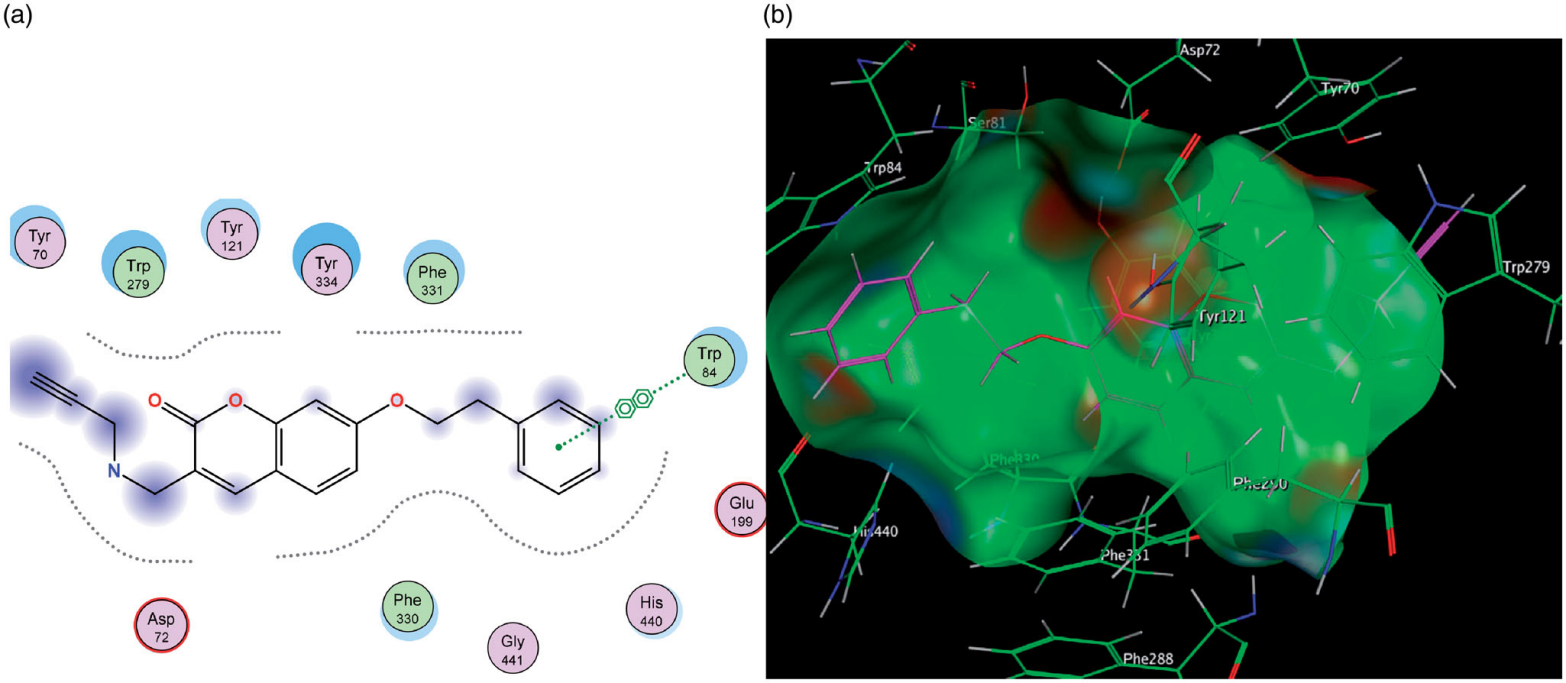
(a) Diagrammatic representation of 3-propargylamine derivatives’ lack of binding interactions with AChE. The π–π stacking with Phe 330 is diminished. (b) 3-Dimensional representation of docking simulation (bottom) with **6** indicated in pink and AChE’s active site’s amino acids indicated in green. The coumarin portion now points away from the CAS and does not partake in significant interactions within the active site.

The improved inhibitory capacity of compounds **4** and **5** can be attributed to how the -CH_2_Br group forms π–H interactions with Trp 84 and the π–π stacking between the aromatic region of the coumarin and Phe 330 (Figure 10). Both of these are key residues for substrate binding in the CAS. It can be hypothesised that the electron-withdrawing effect caused by bromine may have led to more stable interactions with the aromatic Trp 84 residue and was responsible for these compounds’ superior inhibitory activities. Substitution with propargylamine in the same position in derivatives **6** and **7** resulted in added length to the molecules, however, this altered the conformation of the compounds in the active site. As a result, the binding interactions which had been demonstrated with **4** and **5** were disrupted (Figure 11).

#### 3.2.6. Cytotoxicity studies

**Figure 12.**
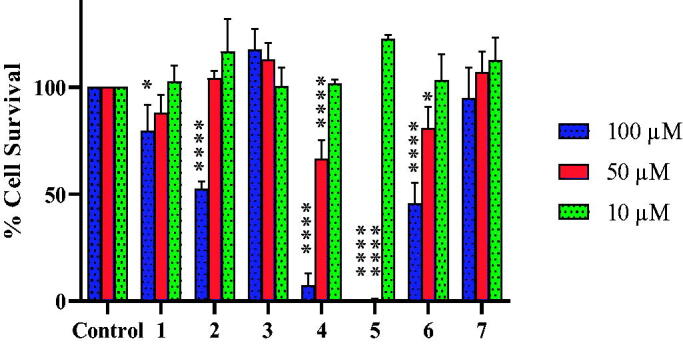
Percentage of cells viable following incubation with test compounds relative to a control of untreated cells. Each bar represents the mean percentage survival and SD (where *n* = 3, three fields per repeat). The data was subjected to an ANOVA statistical analysis and significance was defined as [*p < 0.05 and ****p < 0.0001] when comparing the compounds’ means to the negative control.

The MTT (3-(4,5-dimethylthiazol-2-yl)-2,5-diphenyltetrazolium bromide) assay was used to assess the cytotoxicity of test compounds following their exposure to the SH-SY5Y neuroblastoma cells at concentrations of 10 hx00B5;M, 50 µM, and 100 µM using neuroblastoma SH-SY5Y cells. The results show that in general, the exposure to the test compounds led to a concentration-dependent decrease in the SH-SY5Y cell viability (Figure 12). With the majority of the compounds, this decrease in viability was not statistically significant (*p* > 0.05) at the 50 µM and 10 µM concentrations. At 10 µM the compound’s cytotoxic effect is diminished and can be seen to cause a slight favourable increase in cell viability similar to the untreated control. The 3-bromomethyl derivatives **4** and **5** displayed the most concentration-related cytotoxicity, with a significant reduction in cell viability at 50 µM and 100 µM. Compound **4**’s profile was significantly better with 65% and 7% survival at 50 µM and 100 µM, respectively, compared to a 0% survival rate at the same concentrations for **5**. The 3-bromomethyl substitution can be assumed to underpin this cytotoxicity and this is in agreement with previous studies describing the general cytotoxicity of halogen-containing coumarin derivatives^53^^,^^54^. This cytotoxicity is mitigated at the lower 10 µM concentration.

#### 3.2.7. Neuroprotection studies

The neurotoxin 1-methyl-4-phenyl pyridinium (MPP+) is widely known to induce an apoptotic cascade in neurons and is thus used to induce neurodegeneration in various *in vitro* and in vivo models of NDs^55–58^. In this study, the neuroprotective nature of compounds was determined using an *in vitro* MTT assay based on their ability to rescue SH-SY5Y neuroblastoma cells from this MPP^+^ induced in neurotoxicity.

**Figure 13.**
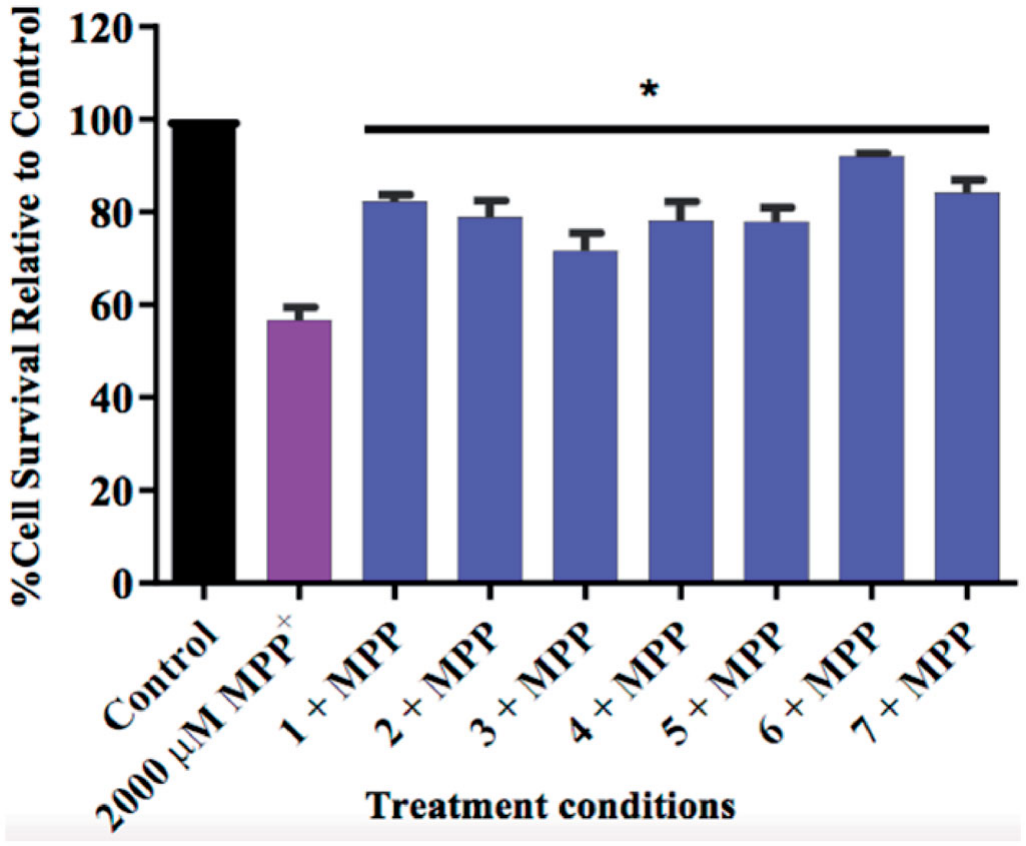
Bar graph comparing the effect of the test compounds (10 µM) on MPP^+^-induced toxicity in SH-SY5Y cells. The cell viability count of the untreated control was defined as 100%. Error bars represent the standard deviation of the mean data. Statistical significance (*p < 0.05) when compared to the MPP^+^ only treated cells (purple bar) is indicated on the graph.

The SH-SY5Y cells were pre-treated with 10 µM of the test compounds for 2 h followed by the addition of 2000 µM of MPP^+^. The 10 µM concentration was selected as the compounds did not cause any cytotoxicity at this concentration, thus it would be a fair representation of their neuroprotective effect. The treated cells displayed higher survival counts compared to the MPP^+^-treated cells (Figure 13), which indicates that the compounds provided neuroprotection to the cells. The cells exposed to the test compounds increased significantly increased (*p* < 0.05) the survival rate (15–36%) when compared to the MPP^+^ only treated group. The nature of the moiety at position 7 does not seem to influence this neuroprotection greatly, as there was no significant trend observed between the 7-benzeloxy and 7-carbamate derivatives. However, as expected, the propargylamine derivatives exhibited the highest neuroprotective capability as reported in the literature^24^^,^^56^^,^^57^. Cell lines treated with compounds **6** and **7** had 92% and 85% survival rates respectively, further demonstrating the moiety’s significance in neuroprotection.

The general neuroprotective profile of the compounds may also suggest that the observed neuroprotection is independent of their enzyme inhibitory capabilities, as all compounds performed fairly similarly despite having different enzyme inhibitory profiles. As described in the literature, coumarin derivatives may possess inherent anti-inflammatory, anti-apoptotic and antioxidant effects which, together with the MAO-inhibition effects of the test compounds may synergistically counter the MPP^+^ induced apoptosis^29^^,^^35^^,^^36^^,^^58^. The results give an indication that the compounds would be tolerated well by neurons at or below the 10 µM concentration and most importantly, potentially ameliorate the neurodegeneration associated the with the progression of AD, and/or other neurodegenerative disorders.

The accumulation of extracellular Aβ plaques has been marked as one of the pathological hallmarks of neurotoxicity associated with AD^59–61^ and coumarin moieties have previously shown to inhibit Aβ plaque formation and Aβ induced neurotoxicity^62^^,^^63^. To further explore the neuroprotective potential of the propargylamine-containing coumarin derivatives **6** and **7**, their ability to curb neurotoxicity induced by the amyloid-β (Aβ) protein was thus assessed according to the method described in the experimental section. In order to evaluate these compounds, the Aβ 25–35 peptide fragment was used to induce neurotoxicity in SH-SY5Y cells. Aβ 25–35 has been reported to be the actual biological active site of Aβ responsible for the peptide’s neurotoxic ability and has been used in similar models of neurodegeneration in previous studies^11–14^.

The exposure of Aβ 25–35 on the SH-SY5Y cells for 24 h exhibited significant (*p* < 0.01) detrimental effects, when compared to the control, with a loss of cell viability of between 30% and 45% in five independent experiments conducted in triplicate. This reduction in cell viability in SH-SY5Y cells after Aβ 25–35 treatment was also reported in previous studies^64^^,^^65^. As the addition of Aβ 25–35 in SH-SY5Y cells precipitates apoptosis^66^^,^^67^, the neuroprotective activity of **6** and **7** on mitigating cellular apoptosis was examined at concentrations between 10 µM and 100 µM. Unfortunately, the compounds, at all concentrations, did not show any significant ability to mitigate the cytotoxic effects caused by Aβ 25–35. The significant neuroprotection observed in the MPP^+^ assay, however, highlights the need for further neuroprotective and mechanistic studies. This will shed light on the potential of these compounds as neuroprotective agents in not only AD but other neurodegenerative disorders such as Parkinson’s disease (PD) as well.

#### 3.2.8. In silico pharmacokinetic and drug-likeness evaluation

In addition to displaying the relevant biological activities, it is equally important for ideal drug candidates to have low toxicity and access to their therapeutic target. Computer models used in the discovery phase with online tools such as SWISSADME are used to provide an early estimation of drug candidates’ ADME, drug-likeness, and pharmacokinetic properties. This decreases the fraction of pharmacokinetics-related failure in the clinical phases^68^^,^^69^. The most promising drug candidates from this study, compounds **6** and **7**, were assessed for their drug-likeness and pharmacokinetic profiles using the SWISSADME web tool.

**Figure 14.**
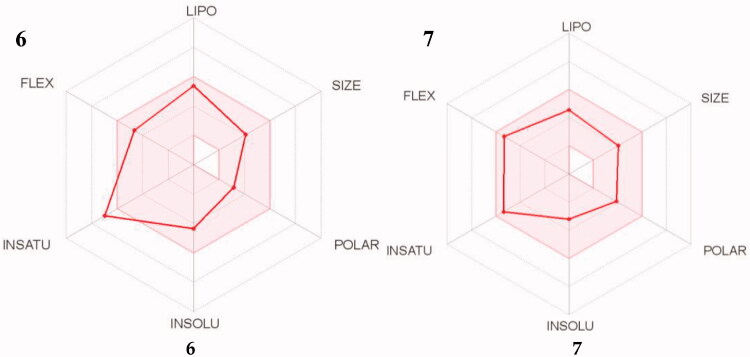
The simulated oral bioavailability of compounds **6** and **7** represented as a radar chart.

A bioavailability radar (Figure 14) was generated, which provides a graphical snapshot of the drug-likeness parameters of an orally available bioactive drug. A range for each parameter was defined and depicted as a pink area on the radar plot. The parameters are defined as lipophilicity (LIPO; −0.7 ≤ XLOGP3 ≤ +5.0); size (150 ≤ MW ≤ 500 g/mol); polarity (POLAR; 20 ≤ TPSA ≤ 130 Å2); solubility (INSOLU; log S ≤ 6); saturation (INSATU; fraction of sp3 hybridised carbons ≥ 0.25) and flexibility (FLEX; ≤ 9 rotatable bonds). For a molecule to be considered drug-like its radar plot has to fall entirely within the defined range^70^.

The compounds were found to be within the desirable pink area, barring compound **6**’s high INSATU value. In spite of this, further *in silico* simulations showed that both compounds have high predicted intestinal absorption and BBB (blood–brain barrier) permeability (Table 3)^71^. BBB permeability is of utmost importance to the agents’ mechanism of action as they need to cross the barrier to inhibit the action of enzymes in the brain as well as offer neuroprotection to affected cells. The Abbot Bioavailability Score predicted the compounds to have high oral bioavailability and they are non-substrates of the permeability glycoprotein (P-gp). P-gp is an efflux pump in the GIT which pumps out xenobiotics (including drugs) away from the BBB and intestinal epithelium, reducing their bioavailability^72^. Finally, the compounds were found to comply with the computational filters developed for identifying drug-like entities by pharmaceutical companies, Lipinski (Pfizer), Ghose (Amgen), Egan (Pharmacia), Veber (GSK), and Muegee (Bayer)^73^^,^^74^. Based on these results the compounds could serve as promising lead compounds for AD.

**Table 3.** In *silico* drug-likeness properties of compounds **6** and **7**

Compounds	BBB^a^	GIA^b^	P-gp^c^		Drug-likeness					
					Lipinski	Ghose	Veber	Egan	Muegge	Bio. Score^d^
**6**	Yes	High		No	Yes	Yes	Yes	Yes	Yes	0.55
**7**	Yes	High		No	Yes	Yes	Yes	Yes	Yes	0.55

^a^BBB: Blood brain barrier permeant; ^b^GIA: Gastrointestinal absorption; ^c^P-gp: P-glycoprotein substrate; ^d^Bio. Score: Abbot bioavailability score (probability of >10% bioavailability).

## 4. Conclusions

The objectives of this study were to synthesise a number of neuroprotective coumarin derivatives substituted with various privileged moieties for potential cholinesterase and MAO inhibitory activities. The compounds were synthesised and characterised and biological evaluations showed that despite their lack of cholinesterase inhibitory activities, they are in general potent, selective and reversible inhibitors of MAO-B and offer excellent *in vitro* neuroprotection in the MPP^+^ model with low cytotoxicity profiles. Especially compounds **6** and **7** displayed the greatest potential as MTDLs and this may be attributed to their inclusion of the propargylamine functional group. *In silico* studies using the online SwissADME tool predicted that the compounds should have acceptable pharmacokinetic and drug-like properties. Additional work is suggested for **6** and **7** to further explore their neuroprotective potential in AD and related neurodegenerative disorders, such as PD.
